# A 5G-Enabled Smart Waste Management System for University Campus †

**DOI:** 10.3390/s21248278

**Published:** 2021-12-10

**Authors:** Edoardo Longo, Fatih Alperen Sahin, Alessandro E. C. Redondi, Patrizia Bolzan, Massimo Bianchini, Stefano Maffei

**Affiliations:** 1 Dipartimento di Elettronica, Informazione e Bioingegneria, Politecnico di Milano, 20133 Milano, Italy; edoardo.longo@polimi.it (E.L.); fatihalperensahin@gmail.com (F.A.S.); 2 Polifactory-Dipartimento di Design, Politecnico di Milano, 20133 Milano, Italy; patrizia.bolzan@polimi.it (P.B.); massimo.bianchini@polimi.it (M.B.); stefano.maffei@polimi.it (S.M.)

**Keywords:** Smart Campus, smart waste management, waste classification, multi access edge computing

## Abstract

Future university campuses will be characterized by a series of novel services enabled by the vision of Internet of Things, such as smart parking and smart libraries. In this paper, we propose a complete solution for a smart waste management system with the purpose of increasing the recycling rate in the campus and provide better management of the entire waste cycle. The system is based on a prototype of a smart waste bin, able to accurately classify pieces of trash typically produced in the campus premises with a hybrid sensor/image classification algorithm, as well as automatically segregate the different waste materials. We discuss the entire design of the system prototype, from the analysis of requirements to the implementation details and we evaluate its performance in different scenarios. Finally, we discuss advanced application functionalities built around the smart waste bin, such as optimized maintenance scheduling.

## 1. Introduction

The Internet of Things vision is becoming a reality, transforming the way we live and interact with the environment. Many conventional places are acquiring smart characteristics thanks to a multitude of small, low cost and connected computing devices able to sense, process and communicate data from the environment to cloud/Internet services. Smart Cities and Smart Buildings, to name a few, are all different realizations of such a vision and are nowadays of great interest to academic researchers as well as having great potential for the industrial world. Smart Campuses are of particular interest in this scenario, and they can be seen as the perfect place for initial steps towards the realization of large-scale projects targeting Smart Cities [1]. Indeed, university campuses mimic cities in many aspects: they generally extend on a vast urban area, they are composed of many buildings of different types (administrative buildings, research laboratories, classrooms, residences, bar/restaurants) and populated by different types of people (students, teachers, administrative and technical staff, etc.). At the same time, the management is somehow more flexible than what is found in proper cities and municipalities since universities are by nature more open at accepting innovations and new technologies, even if still not completely mature. Several solutions have been recently proposed in association with the concept of Smart Campus [2]: smart parking systems [3,4], microgrids [5], smart libraries [6,7], systems for classroom monitoring and occupancy estimation [8,9] as well as sustainable solutions [10,11], are all examples of smart applications implemented in university campuses.

One area that received particular attention in the last decade is the efficient management of university solid waste (USW) [12]. Recycling such waste is crucial from several points of view: from an economic perspective, turning solid waste into a resource is fundamental to the realization of a circular economy, where one industry’s waste becomes another raw material. At the same time, efficient and sustainable management of waste helps reduce health and environmental problems: recycling materials helps cut emissions from landfills and from new extraction/processing sites, and mitigates environmental issues such as water/air pollution and littering.

Several works in the literature have addressed the analysis of how much waste is produced in a university campus, with estimates ranging from 50 to 150 g/user/day (i.e., 20–50 Kg per user each year) [13,14,15]. Considering that, according to recent statistics [16], each person in Europe produce half a tonne of municipal waste per year, USW alone account for about 1 tenth of the total waste produced in cities.

Moreover, some studies [17,18] have analysed the composition of waste produced in university campuses, concluding that the majority of USW is composed of organic waste suitable for composting, followed by recyclable materials such as plastic, glass and paper. As different types of waste require different recycling processes, segregating and separating waste at its source is key to the effective management of the recycling chain. While industrial waste is generally treated with large-scale segregators, the task of waste separation is much more challenging at the municipal or campus level, as it is solely based on the goodwill of people and the level of readiness of the recycling infrastructure available.

To facilitate the separate collection of waste starting from the beginning of the sorting chain, that is, from public waste bins, information technologies may come to help. In particular, embedding different types of sensors and actuators into such waste bins, connecting them to the Internet and driving them through intelligent algorithms (i.e., following the vision of the Internet of Things (IoT)) may give an incredible boost to the recycling performance.

Motivated by these reasons, this paper extends our previous paper [19] and describes the realization of a complete solution for the efficient management of USW. The key building block of our proposal is a novel prototype of a Smart Waste Bin (SWB), a smart object able to automatically sort different types of trash directly at the place of generation using a multi-sensor approach, thus easing the management of the entire trash cycle. Peculiar features characterize the system: the SWB adopts a hybrid scalar/visual sensor waste classification algorithm that allows for accurate waste recognition as well as an innovative dual-motor design for automatic waste segregation. Moreover, the SWB and the management system designed around it are integrated with the recently introduced 5G networking architecture, particularly for what concerns the advantages of using a Multi-access Edge Computing (MEC) server. Indeed, the intelligence driving the SWB resides at the edge of a 5G cellular network, rather than in a cloud server or locally on the object itself. This approach brings several benefits, such as reduced delay in waste recognition and reduced energy consumption, making the SWB more appealing to everyday use.

In detail, the contributions of this work are the following: first, we illustrate the design and implementation of the smart waste bin, detailing the steps made for its creation from the analysis of the requirements to the physical realization of its external and internal parts. Second, we give details on the algorithms governing the SWB, including the main functioning logic as well as the multi-sensorial artificial intelligence used for recognizing and sorting different types of trash. For the latter, we propose different ways of fusing information coming from the different sensors, evaluating the performance obtained. Third, we evaluate a fully working prototype of the SWB in different scenarios, showing through experiments on a real 5G network that moving the artificial intelligence on the MEC is beneficial under both latency and energy consumption perspectives. Finally, we showcase the potential of a management system built around a multitude of (simulated) smart waste bins, allowing for, e.g., easy and optimized maintenance.

The remainder of this paper is structured as follows: Section 2 briefly reviews the main related works in the field of smart waste management. Section 3 details the physical realization of the proposed smart waste bin prototype, focusing on the main working logic and the offered functionalities. Section 4 provides a detailed description of the hybrid scalar/visual machine learning algorithm used to perform waste classification and evaluates the performance obtained when such intelligence is run locally on the SWB, in the Cloud or on a 5G MEC. Section 5 focuses on the management backend server and the advanced features offered by the provided user application. Finally, Section 6 concludes the paper.

## 2. Related Work

Facilitated by the wide commercial availability of low-cost sensors, microcontrollers and communication modules, several research works focusing on prototyping smart waste management systems have appeared in the last few years.

A class of these works focus mainly on monitoring the amount of trash and the fill level of waste bins in order to send alerts and optimize the emptying procedures [20,21,22]. Generally, ultrasonic sensors are used to estimate the fill level by measuring the distance from the lid on top of the bin to the trash in the compartment. Sometimes a load cell sensor is incorporated at the bottom of the bin to measure the weight of the waste [23,24]. As an example, in [25] authors propose a system with ultrasonic sensors connected to a Microcontroller Unit (MCU) that sends an SMS message to the municipality if the waste level is above a certain threshold. Knowing the waste levels and the locations of the corresponding bins, the routing and scheduling of the garbage picking procedures can be optimized; as a result, authors claim that the service cost can be cut by 50%.

The second class of works focus on techniques for recognizing and sorting different types of trash, with several approaches. Some works use scalar sensors, such as electromagnetic sensors (capacitive or inductive sensors), which can be utilized for detection of nonferrous metal fractions based upon electrical conductivity of the sample [26,27]. Alternatively, photoelectric sensors (obtained coupling a Light Emitter Diode (LED) source and a photodiode as a receiver) can be used to recognize the type of material (especially in presence of transparent wrappings) [28]. Other works focus on Radio-frequency identification (RFID) technology to sort the different categories of waste, assuming that each piece of trash is equipped with a smart RFID tag containing the information on the particular type of material [29,30].

With the success and popularity of machine learning, and in particular of Convolutional Neural Networks (CNN) in the field of computer vision, a considerable amount of works tackle the problem of image-based waste recognition [31,32,33,34]. A common approach is to use already existing CNN models (pre-trained over very large image databases, such as ImageNet [35]), which are known to provide excellent results in terms of image classification (e.g., AlexNet [36] or VGG16 [37]), and fine tune their last layers of the neural network with datasets containing images of pieces of trash [38]. All these works report excellent performance in the task of trash classification, reporting accuracies generally above 90% when four target classes of glass, paper, metal and plastic are concerned.

Some works also propose prototypes not only to recognize different pieces of trash, but also to move them in proper compartments after recognition. The operation is typically performed through the use of Direct Current (DC) or stepper motors [39,40]. As an example, in [41] waste is placed on a conveyor belt and classified in different categories via image-based recognition and a trained CNN. After classification, an automatic hand hammer is used to push the waste into a specifically labelled bucket.

For what concerns communication technologies, most of the aforementioned works contemplate the use of radio technology to communicate application data such as the bin fill levels or other information to a remote management server. Often, a GSM module is used [23,24,25], although recent works explored the possibility of using other types of communication such as LoRa/LoRaWAN [42,43].

This paper proposes a complete solution for waste management that comprises most of the features encountered in the recent literature. The proposed Smart Waste Bin offers accurate waste classification through a hybrid scalar/visual sensor system, as well as automatic waste segregation with an innovative dual-motor setup and waste level tracking. In addition, the entire system makes efficient use of 5G connectivity and the availability of MEC technology to increase recycling rates while providing reduced operation costs, response time and energy consumption.

## 3. Building the Smart Bin

### 3.1. Requirement Analysis

**Figure 1:**
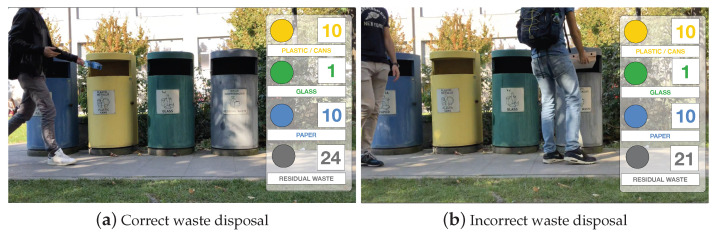
Two frames of the recordings used for analysing the student’s behaviour. The average interaction time is estimated from the video.

**Figure 2:**
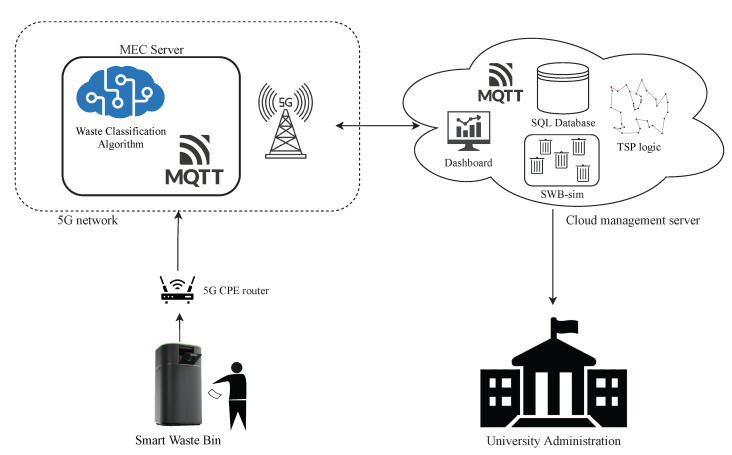
Overview of the Smart Waste Management System: the Smart Waste Bin leverages 5G MEC architecture to accurately classify trash in order to automatically segregate it. Usage data from the smart waste bin is also transmitted to a central backend server, which allows the university administration to provide optimized waste management.

Before designing the system, we conducted an analysis to understand (i) how people interact with the traditional waste bins currently available in the campus premises and (ii) what is the composition of the waste produced, two pieces of information that are key for building an effective yet user-friendly prototype. The analysis was conducted in the Bovisa Campus of Politecnico di Milano university, which hosts departments and classrooms for both the Engineering and Design schools and hosts roughly 10,000 people considering students, faculty and administrative staff. For one week, we filmed the behaviour of people during the lunchtime break (12:30–13:30), collecting statistics on the type of trash produced as well as studying the behaviour of each person when handling the trash in front of the existing waste bins. The area analysed is an area generally used by students for consuming lunch. Two trash collecting points are present in the area, both equipped with four coloured bins collecting different types of trash according to the regulation of the municipality of Milan (paper, plastic/aluminium, glass, unsorted trash) (Figure 1). We observed that the most recurring behavior of a person after lunch is to collect all pieces of trash, move to one of the waste collecting points and then manually sorting all pieces of trash in the correct bins, one at a time. Another observed behavior consists of throwing all the different pieces of trash in the unsorted bin. Although such a latter behavior happens less frequently, it is detrimental for recycling purposes. In total, we analysed about 400 interactions between humans and trash bins: the average amount of time spent by the first group of users, the ones sorting the trash in the correct bins, is 5.3 s. The composition of the waste produced is observed as it follows: 24% plastic/aluminium, 22% paper, 2% paper and 52% residual waste (unsorted). Such percentages are in line with other studies conducted in university campuses [18]. Based on such observations, we designed a Smart Waste Bin able to (i) accurately classify and segregate trash while requiring minimal effort to the users and (ii) keep the required interaction time below the average observed during the requirement analysis. The realized bin is the central element of a more general Smart Waste Management System, illustrated in Figure 2, and detailed in the following sections.

### 3.2. Prototype Design

**Figure 3:**
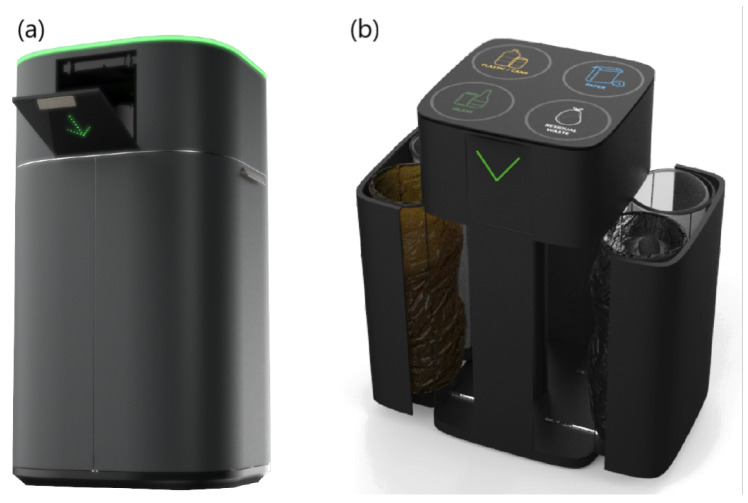
Three-dimensional (3D) render of the Smart Waste Bin: (**a**) on top, the lid of Waste Disposal Unit with LED feedbacks, (**b**) on the bottom, the garbage unit in its open position, showing the internal bin bags.

**Figure 4:**
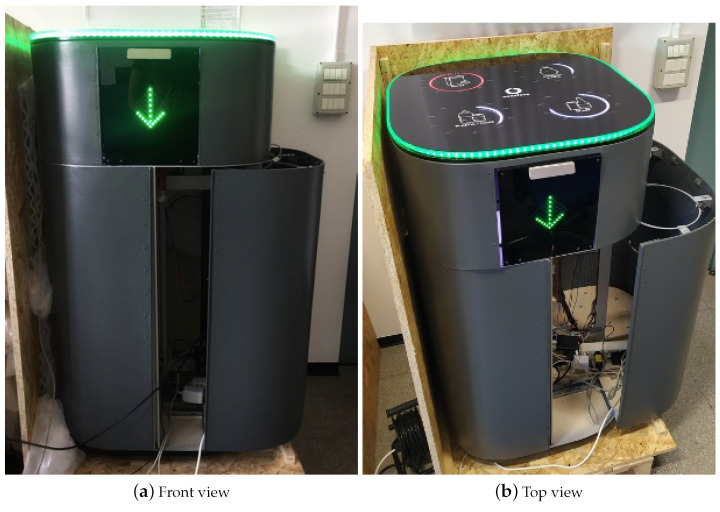
Smart waste bin: realized prototype.

The proposed Smart Waste Bin (SWB) is composed of a unique solid body measuring 90 cm in height, with a diameter of 62 cm. The external body is digitally fabricated with a large size FDM (Fused Deposition Modeling) 3D printer, using a thermoplastic material. A washable protective varnish is applied on the whole exterior prior to the final colouring process. Figure 3 shows a digital render of the prototype, while Figure 4 shows the final realized version. The 3D-printed body hides an aluminium structure, which gives solidity to the entire prototype and is used for supporting all the hardware and the electronics needed, as well as the Garbage Unit (GU). The GU contains four circular aluminium structures, used for holding four standard 110 L bags for collecting glass, paper, plastic/metal and residual waste. We opted to maintain the same type of garbage bags already used for traditional bins in the campus for all type of waste, although the requirement analysis clearly showed different usages among the four different type of waste, in order not to modify the supplying operations of the waste management service of the campus and facilitate a transition between already existing bins and smart bins. To ease the tasks of garbage bag replacement, cleaning and other maintenance activities, the entire GU can be easily opened through sliding guides placed at the bottom of the SWB (as shown in Figure 2, right).

**Figure 5:**
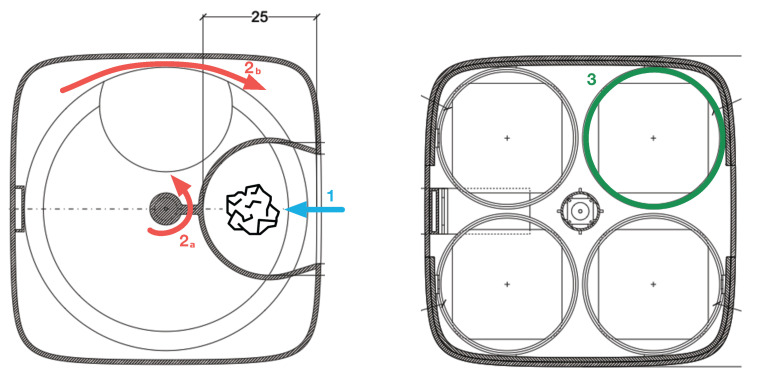
Waste disposal unit. (1) A piece of trash is inserted into the SWB and recognized. (2a) The semicircular structure acts as a mechanical arm and moves the trash towards the correct bin. Concurrently (2b), the shelf moves to let the trash fall into the correct bin in the garbage unit (3).

A convenient flap door is placed on the front side of the Smart Bin, easily accessible through a metal handle mounted on its top. The door embeds a LED matrix, covered with a laser-cut semi-transparent plastic material, which is used to signal if the SWB is correctly functioning (with a green arrow) or not (with a red cross). In the latter case, an automatic lock avoids opening the door. In normal conditions, opening the door reveals the Waste Disposal Unit (WDU), where objects to be thrown away are deposited and eventually recognized, one at a time. The user deposits a piece of waste in the WDU, which contains a rotating circular shelf with an aperture surrounded by a semicircular structure connected to a couple of servo motors (Figure 5). This area is used for taking measurements from the piece of trash using a hybrid scalar/visual sensor system, which are subsequently fed to a waste classification algorithm (detailed in Section 4). After the waste is recognized in one of the four trash classes, it is automatically moved in the proper bag, thanks to the servo motors.

The top part of the SWB is composed of a plastic surface that protects four circular LEDs indicators and a LED string, which are used as visual feedback for the user. The surface is fabricated starting from an anti-scratch piece of semi-transparent rigid plastic, which is later processed with a laser cutting machine and then engraved to make the LEDs visible. The four circular LEDs on the top are used to indicate the fill levels of each bag, respectively, in white from 0% to 99% and in red when the 100% is reached. The LED string contouring the top part is again used to signal the operational status of the bin with the same colour code of the front LED matrix: static green indicating that the SWB is ready for collecting a piece of trash, blinking green for the trash processing phase, and red in case of malfunctioning or if the bin bags are full.

### 3.3. Sensors and Actuators

The Smart Waste Bin exploits heterogeneous sensors and actuators for recognizing and sorting the trash, respectively. Such sensors and actuators, as well as the logic of the system, are controlled by a Raspberry Pi 3 Model B+, which is attached to the internal aluminium structure of the SWB and directly connected to a power socket. For communication with external services, the Raspberry Pi is connected through the internal WiFi interface to a Huawei 5G CPE router provided by Vodafone Italia S.p.A, as explained in Section 4.3.

#### 3.3.1. Waste Sensing Module

The waste classification algorithm, explained in Section 4, is based on a hybrid scalar/visual Waste Sensing Module (WSM) which exploits different types of sensors. The main tasks of the WSM are (i) detecting when an object has been inserted into the WDU of the bin and (ii) acquiring measurements from the piece of waste for subsequent analysis and recognition. For what concerns the waste detection task, the WDU is equipped with a pair of Time-of-Flight (ToF) VL53L0X distance sensors, which are able to accurately detect whether or not an object is in the area and, subsequently, trigger the sensing process. Upon detection of a new object, the WSM leverages the following sensor for gathering measurements:

- *Inductive Sensor:* an LJ12A3-4-Z/BX sensor is attached to the bottom of the shelf in the WDU, used for non-contact detection of metallic objects. The detection range limit of this sensor is about 5mm: therefore, it is placed in the center of the WDU, which is curved to facilitate objects to slide towards the sensor.
- *Capacitive Proximity Sensor:* such type of sensors are generally used for non-contact detection of both metallic and nonmetallic objects. Here, we used an LJC18A3-H-z/BX sensor placed close to the inductive sensor. The detection range of the sensor is about 10 mm.
- *Photoelectric Sensors:* three couples of photoelectric emitter/receiver are attached at the two opposite sides of the WDU, in through-beam configuration. The emitters are standard LEDs, while we used BPW21 photodiodes as receivers. Such sensors may be used to detect transparent materials such as plastic or glass.
- *Camera:* on top of the WDU, a Logitech C920 wide-angle camera is placed at 45 cm from the surface, with an inclination of 30 degrees. The camera is configured to acquire images at a resolution of 320 × 240 pixels.

The data acquired by such sensors is then passed to the waste classification algorithm, which is detailed in Section 4.

#### 3.3.2. Automatic Waste Segregation

Trash segregation is obtained through a pair of servo motors, which allow for precision control of the movement and rotors position. The two motors are located in the central spindle of the bin, one on top of the other and allow to move a piece of trash in the proper bag. One motor controls the plastic shelf rotation in the WDU, while the second is attached to the semicircular structure. Disposal of a piece of waste happens in two steps: first, the shelf and the semicircular structure rotate in the same direction so that the piece of trash is located on top of the right bin. Then, the shelf rotates in the opposite direction so that its aperture let the piece of trash fall into the bin (Figure 5). Both motors are wired to the Raspberry Pi and controlled through the GPIO pins. In order to ensure the correct positioning of the two motors, they are automatic calibrated during every boot of the SWB thanks to specific magnets located on the motors’ hardware.

#### 3.3.3. Fill Levels Engine

**Figure 6:**
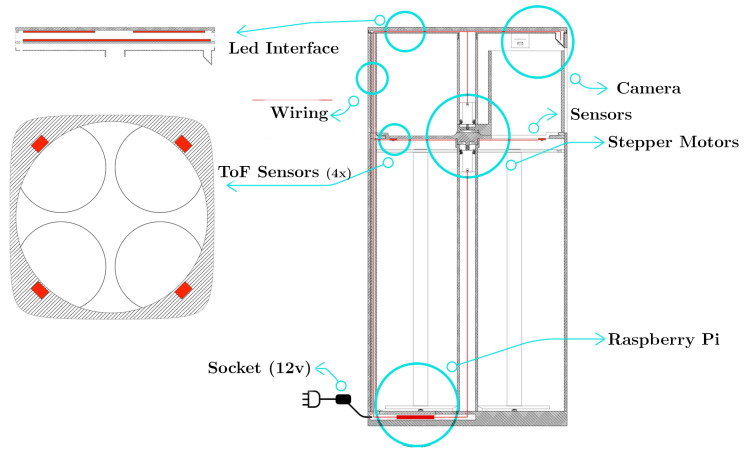
SWB vertical section and internal details.

Each bag in the garbage unit is equipped with a Time-of-Flight (ToF) VL53L0X distance sensor, similar to the one used in the Waste Sensing module. Thanks to a laser, the sensors can accurately measure the distance between the top of the GU (Figure 6 and the garbage inside the correspondent bin bag, providing the estimated fill level of each trash bag. Then, the fill level is used as user feedback displaying the percentage level on the upper surface of the smart waste bin through LED strips, and transmitted to a remote server for advanced functionalities and management purposes.

### 3.4. Standard Operating Procedure

**Figure 7:**
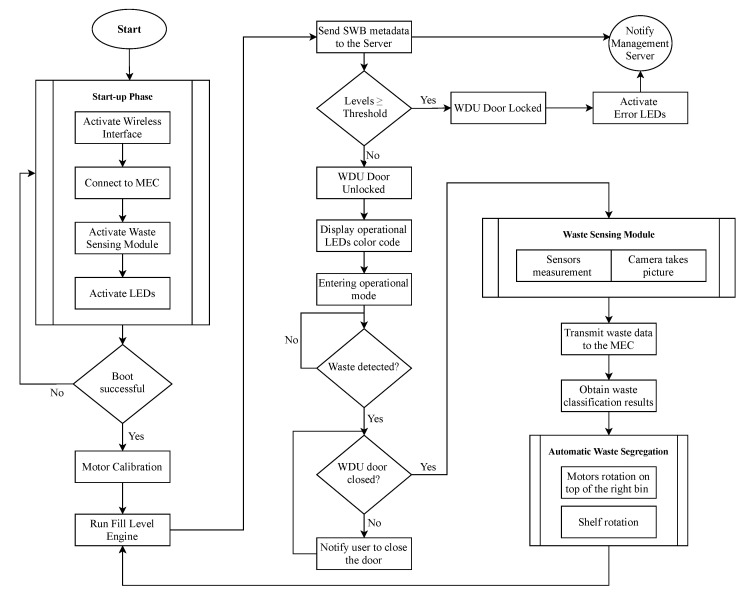
Functional flow diagram of the smart waste bin.

Figure 7 illustrates the functional flow diagram of the smart waste bin. Upon activation, the smart waste bin performs the following operations:

1. *Start-up routine*: during this phase, the firmware executes a series of checks for all the sensors and actuators, as well as for the wireless connectivity with an external server. If all the checks are passed, the SWB can be started; otherwise, the flap door is locked and all LEDs are turned to red color.
2. *Motors synchronization and calibration*: after the start-up, the motors that control the automatic waste segregation need to align with the waste disposal unit to ensure correct disposal of trash. Such regulation allows the motors to set their starting position and subsequently compute the positions (in terms of degrees of rotation) of the four trash bags. For this purpose, the motors perform one complete 360 degrees start-up spin: we use a magnet and a Hall effect sensor to mark the starting position of both the shelf and the semicircular structure, thus calibrating the system.
3. *Fill Levels Engine*: after the controlling operations, the SWB verify that it has enough room to store new pieces of trash, using the Fill Levels Engine. The current level of each bag is estimated and transmitted via MQTT to an external server. The topic used for such signalling is smartbin/swb_id/fle/material where swb_id and material are the strings controlling the SWB identifier and the waste material corresponding to the sensed bag. If the levels exceed a specified threshold (75% in our case), the waste management administrator is promptly notified, in order to empty the bin before it can saturate the bag size. Moreover, when one or more bags fill levels reach 100% of the capacity, the bin activation is interrupted, the door is locked, and all LEDs are turned red, waiting for an operator to take action.

When all the operations above are completed, the smart waste bin enters the idle state and the LEDs on the top (as in Figure 4a) become green, indicating it is ready to accept recycling items. The operations are as follows:

4. *Waste insertion*: when the SWB is active, a user willing to throw a piece of trash can open the lid of the waste disposal unit, insert an object on the shelf as in Figure 5(1) and, finally, close the lid to activate the classification process.
5. *WSM activation*: upon the closure of the WDU, the Smart Waste Bin, thanks to the Time-of-Flight sensors, detects that an object is ready to be analysed. At this point, in order to avoid any interference from outside, the SWB securely closes the lid and activates the waste sensing module, gathering measurements from the sensor as well as taking an image with the installed camera.
6. *Waste Classification and Segregation*: the sensed data is passed to the waste classification algorithm (Section 4), which returns, as a result, the estimated type of the piece of trash. Finally, the motors are activated and the object is moved in its correspondent bag.
7. *Release*: after the object is disposed correctly, the motors come back to the starting position, the fill levels engine is again activated, and the SWB is ready for another operation.

## 4. Waste Classification Algorithm

The smart waste bin implements a hybrid waste classification algorithm that leverages data from both the scalar sensors and the camera installed in the WDU to distinguish the specific type of waste inserted. We train the algorithm to distinguish among four different classes according to the rules of the municipality of Milan: glass, paper, plastic/metal, and unsorted.

### 4.1. Dataset

**Figure 8:**
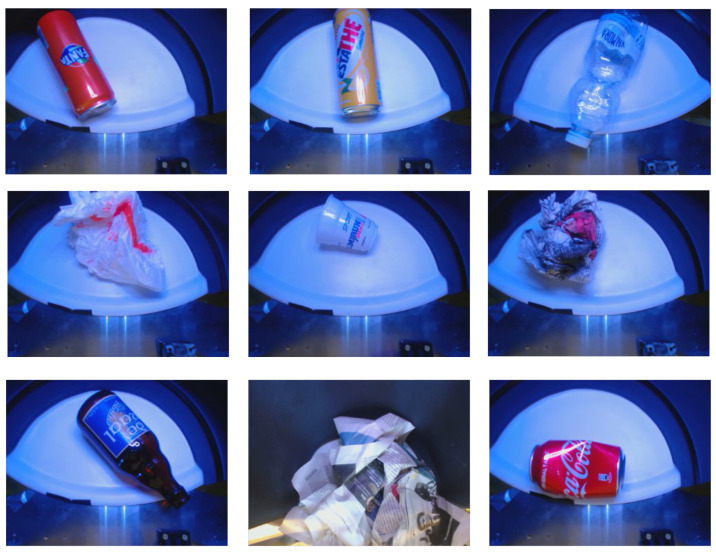
A sample of the pictures used as training dataset. The objects were acquired by the waste sensing module directly on the white shelf of the waste disposal unit.

We created a dataset for training the waste classifier, collecting the most frequent waste items found in our university campus’ bins and surveying the students about the most common garbage objects thrown into the trash. We collected about 65 different waste items, which were inserted into the smart waste bin for data collection. Since waste objects are not always in their pristine forms when being thrown away but are often dirty, distorted, torn, or crumpled, each item was inserted multiple times into the SWB. Each time, we changed the position of the object inside the WDU as well as applied physical deformations to modify its shape. From the initial 65 items, we collected 3125 data observations, each one composed of one image acquired by the camera sensor as well as a vector of measurements collected by the other scalar sensors. Finally, we grouped objects of the same type together: as an example, all different observations of beverages in aluminium cans (e.g., Coke, Fanta Orange, Red Bull) are grouped in the class metal can. After this operation, the final dataset is composed of 40 classes, each one with roughly 80 observations. As a last step, each item in the dataset is labelled with one of the five classes of trash: glass, paper, plastic, metal, and unsorted. We obtained 7 objects in the glass class, 9 in the paper class, 13 for the plastic class, 4 for the metal class and 7 for the unsorted class (see Table 1 for a complete list). Figure 8 shows a sample of the pictures used for the training dataset taken by the bin’s camera, while Table 2 reports the summary statistics for the data gathered by the scalar sensors, divided by class. As one can see from Table 2, the scalar sensors allow capturing some characteristics of specific materials such as the conductivity of metals or the different transparency between paper and plastic. The complete dataset is made publicly available at https://tinyurl.com/SWB-dataset (accessed on 22 May 2021).

### 4.2. Waste Classification

We observe that the type of data returned by the two different types of sensors is very different: the inductive and capacitive sensors return a binary value, the photoelectric sensors return a real value and the camera produces an image. In the following, we will first derive two different classification models, leveraging either the scalar sensor data or the images from the camera. Then, we will explore two strategies to effectively fuse all this information in a single classification algorithm, which differ in terms of *where* data integration happens: at learning time or at prediction time.

#### 4.2.1. Classification from Scalar Data

As a first step, we trained a classifier to leverage the data retrieved by the scalar sensors only. As a preprocessing step, each sensor data was normalized in order to have zero mean and unit variance. We split the available data into train and test subsets, according to stratified *k*-fold cross-validation with k=5. The training data was given as input to a logistic regression classifier, using as labels the object materials. The performance of the resulting 5-class model is evaluated on the test folds, and we report in Table 3 the results obtained in the form of a confusion matrix, considering all test folds. As one can see from the table, waste classification starting from the scalar sensors only allows to already reach a good starting point, with an average accuracy of about 89.6%. Some classes have very high recognition accuracy: indeed, the glass, metal and paper classes are recognized with accuracy higher than 95% given the unique property of the materials and the way they interact with the available sensors (i.e., inductive and capacitive sensors).

#### 4.2.2. Classification from Images

**Figure 9:**
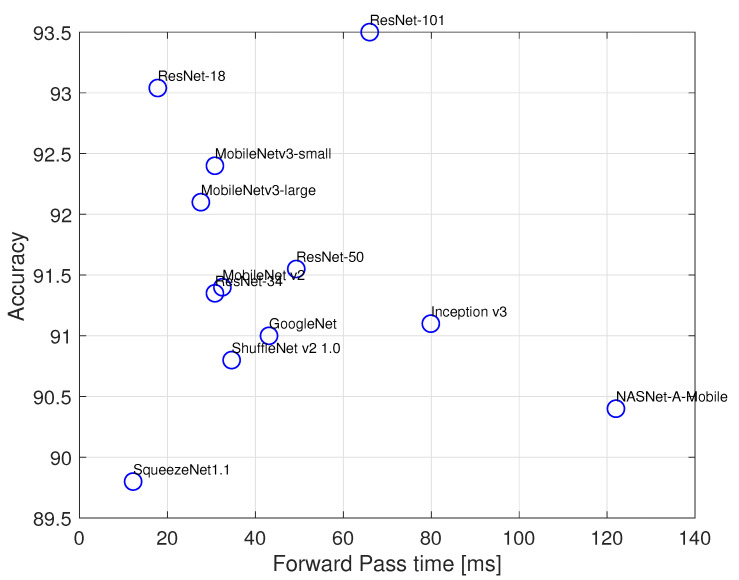
Material class accuracy vs. mean forward pass time.

As a second step, we build an image-based waste classifier. We base our approach on the use of a Convolutional Neural Network (CNN) classifier, thanks to its proven effectiveness in image classification tasks. Training a CNN classifier from scratch, avoiding the issue of overfitting, generally requires a massive amount of training images. Due to the relatively small size of our dataset, we rely on the concept of *transfer learning*: we start from a CNN image classifier pre-trained on the ImageNet dataset [44], and re-train only its last layers on our dataset in order to specialize it to the task of classifying trash. Since each CNN layer learns filters of increasing complexity, the earlier layers learn to detect basic features such as edges, corners, textures whilst later layers detect patterns, object parts, tags, and the final layers detect objects. Therefore, fine-tuning the last layers on our dataset while keeping the previous layers enables us to reach an accurate model without needing a huge image dataset as input. Several pre-trained CNN models, differing in structure (number of layers, number of neurons per layer, etc.) are already available: in order to select the one that best fits our purposes, we performed fine-tuning and studied the resulting model accuracy as well as Single Forward Pass (SFP) time (that is, the time it takes for the CNN to process an image and return the classification result). The following CNN models were considered for comparison: NASNet-A-Mobile, MobileNet-v2, MobileNet-v3-large, MobileNet-v3-small, ResNet-18, ResNet-34, ResNet-50, ResNet-101, GoogleNet, ShuffeNet-v2-1.0, SqueezeNet-v1.1 and Inception-v3. Due to the large variability of the appearance of objects inside each waste material class, tests were performed in the following way: we first split the dataset in train and test folds according to the same 5-fold cross-validation procedure used for the scalar sensor-based classifier. This time, however, we trained the CNNs using the object labels rather than the material labels. At inference time, we mapped back each object to its material class. All tests were performed on an Intel Core i7-6700HQ CPU, equipped with a NVIDIA GeForce GTX 950M GPU, and 16 GB RAM. The accuracies obtained are illustrated in Figure 9: we select the ResNet-18 model as the best compromise between accuracy and SFP time. Table 4 shows the confusion matrix obtained with the fine-tuned ResNet-18 model. As one can see, the average accuracy hits about 93%, with no material class having accuracy higher than 95%.

#### 4.2.3. Hybrid Classification

Looking at the results obtained classifying waste with scalar sensor or image data, it is clear that each method has its pros and cons. Scalar sensor data outperforms image-based classification for some materials (e.g., metal), while image-based classification obtains similar results for each class. In the following, we propose two different strategies to exploit the best features of the two different approaches.

**Figure 10:**
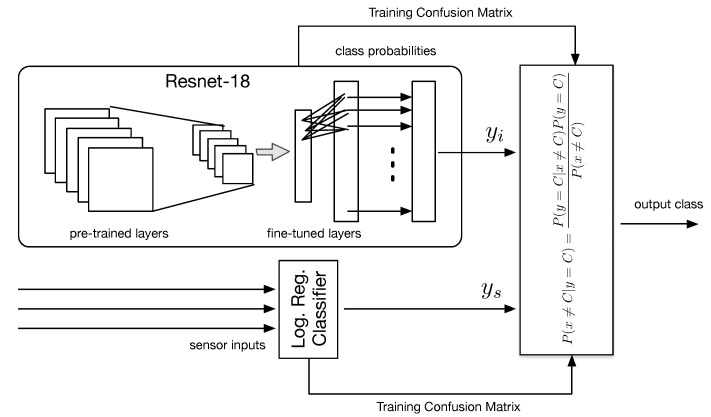
Integration at prediction time.

**Figure 11:**
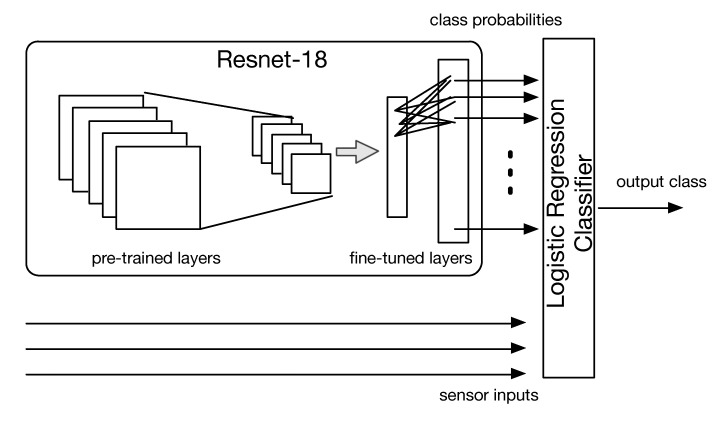
Integration at learning time.

1. *Integration at prediction time:* a first approach consists of running the two classifiers in parallel and then taking a decision considering the lowest (training) classification error (Figure 10). Let *y* be the output of the two classifiers, taking qualitative value *C* = [glass, paper, plastic, metal, unsorted], i.e., the output class. For each classifier, we compute the a posteriori misclassification error probability P(x≠C|y=C), being *x* the true class. To do this, we use the Bayes’ theorem:
  (1) $$P\left(x\neq C|y=C\right)=\frac{P(y=C|x\neq C)P(y=C)}{P(x\neq C)},$$
  where P(y=C|x≠C), P(y=C) and P(x≠C) are the likelihood of misclassification for class *C*, the prior output and the prior class probabilities, respectively. We estimated such quantities from the (training) confusion matrix of each classifier. In case the two classifiers agree on the output class, the method obviously returns the same class *C*; in case the two classifiers disagree, the class *C* having the lowest misclassification error is selected.
  As an example, let $y_{s}$ = plastic and $y_{i}=\mathrm{glass}$ be the output of the sensor-based and image-based classifiers, respectively. Assuming the values contained in Table 3 and Table 4 as the learnt probabilities during training we have:
  (2) $$P\left(x\neq \mathrm{plastic}|y_{s}=\mathrm{plastic}\right)=\frac{0.1038\times 0.303}{0.675}=4.65\%,$$
  while
  (3) $$P\left(x\neq \mathrm{glass}|y_{i}=\mathrm{glass}\right)=\frac{0.1337\times 0.179}{0.825}=2.9\%;$$
  The system will therefore select $y_{i}$ as final class, since its associated error is lower.
2. *Integration at learning time:* A second approach is to train a new classifier, where input features come from all available sensors. To do this, we note that the last layer of the fine-tuned CNN consists of 40 nodes, where each node outputs a value between 0 and 1 that represents the probability that the input image belongs to one of the 40 object classes. We treat such values as new features, which are fed to a regularized logistic regression classifier together with the scalar sensor measurements (Figure 11. The classifier is again trained according to *k*-fold cross-validation using as ground truth labels the waste materials.

The results obtained on the test set for the two strategies are contained in Table 5 and Table 6, for the integration at prediction and learning cases, respectively. As one can see, both approaches allow to increase performance compared to solely using the scalar sensor-based or image-based approaches. In particular, integration at prediction time allows obtaining an accuracy of 96.12%, while the best result is obtained with the integration at learning time approach (97.37%). This is particularly promising, especially to cope with specific waste objects such as that of shattered glass. In this case (fortunately rare, according to our survey) relying solely on an image-based recognition would be very difficult given the high variance associated with images of glass fragments. Indeed, using also scalar sensors in the system may greatly improve the recognition accuracy.

### 4.3. Waste Classifier Location

The hybrid model with integration at learning time has been exported for being tested in three different scenarios, differing in where the classification takes place.

1. *Local recognition:* first, we run the classifier on the Raspberry PI controlling the SWB. In this case, the SWB does not require any connection to an external server as all decisions are taken locally.
2. *Cloud-based recognition:* as a second test, we move the classifier on a cloud-based server hosted on Amazon Web Services EC2, located in Ireland. Data gathered from the WSM is transmitted to a listening process on the server: upon reception, the classifier is run and the response is transmitted back to the SWB. We used SCP to transfer data from the SWB to the server, while the MQTT protocol was used to reply from the server to the SWB.
3. *MEC-based recognition:* finally, we move the classifier on a multi-access edge computing server, provided by Vodafone Italia S.p.A, located in the core Vodafone network in Milan and running an Ubuntu Server machine with the same characteristics of the AWS EC2 instance. Access to the MEC is enabled by using the 5G connection through the Huawei 5G CPE router, which allows for a low-latency and high-bandwidth connection.

For every scenario, we tested the total recognition time of a waste item and the overall energy consumption of the SWB.

#### 4.3.1. Recognition Time

The total recognition time is composed of CNN execution time and the picture transfer time from the Raspberry Pi to the server. (Image acquisition time is assumed constant and thus discarded.) For the local scenario, since the picture is processed internally on the Raspberry Pi, the total time equals the execution time of the CNN, which is around 3 s. For the cloud and MEC server scenarios, the total time also includes the transfer time of the picture from the Bin to the server. In these cases, the Image Acquisition Module of the Raspberry Pi takes a picture of the trash, sends it to the cloud or MEC server using Secure Copy Protocol (SCP); then, the server feeds the picture to the CNN, and the resulting label along with the confidence level is sent back to the SWB as an MQTT publish message. The time measurement summary is given in Table 7.

As one can see, the total time on the Raspberry Pi is 5–6 times longer than the others taking over 3 s due to the low computational power available. Since the cloud and the MEC server have equivalent hardware specifications, the CNN recognition time is identical on the two machines. However, as the MEC server is located closer to the Smart Waste Bin compared to the cloud server, the data transfer time is greatly reduced. For this reason, we can see a clear improvement for the MEC approach in the Average Total Time. In any case, note that the total time is well below the average time of 5.3 s spent with the traditional bins and estimated from the requirement analysis. This means that the use of the SWB speeds up an average interaction with a human, also reducing waste misplacement.

#### 4.3.2. Energy Consumption

**Figure 12:**
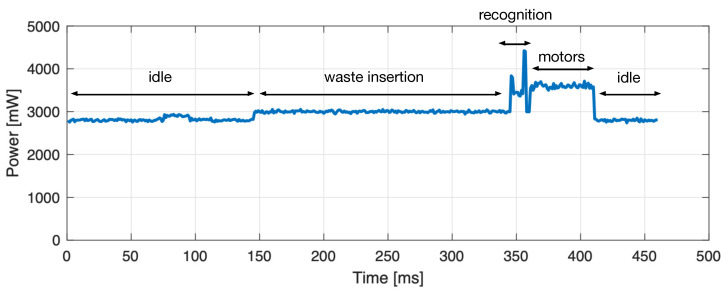
Energy measurement during one normal operation cycle.

To measure the energy consumption of the SWB, we used an Adafruit INA219 High Side DC Current Sensor wired to an Arduino Uno and connected in series to the Raspberry Pi of the SWB. The method calculates the integral of power over the execution time, i.e., the sum of instant power samples taken by the sensor unit, illustrated in Figure 12. Even though the unit continuously takes measurements, since the samples are discrete, the exact energy consumption is not measured but estimated. As one can see in Table 8, the energy consumption reflects the total recognition time. In particular, using the MEC, we can save up to 15% of energy compared to the cloud version.

## 5. Management Application

The smart waste bin collects not only data relative to the waste classification but also a multitude of heterogeneous information such as time and frequency usage, bag filling levels, emptying time. Such additional metadata may be of enormous value for optimizing the waste collection task in a university campus, as well as larger scenarios such as a city. For these reasons, all the information collected by the bin (working status, filling level for each waste class, etc.) are periodically transmitted to a management server, hosted remotely, which stores the data for advanced uses. In the following, we provide a brief description of such a management server: to fully test the functionalities offered, we also provide a Smart Waste Bin simulator (SWB-sim), which allows simulating a multitude of SWB instances, therefore, providing enough data.

### 5.1. Smart Waste Bin Simulator (SWB-Sim)

To overcome the practical issues of physically realizing multiple prototypes, we propose a simulator that virtually creates thousands of bins with different usage profiles, such as frequency of interaction with people and distribution of waste produced. The simulation software is written in Python and replicates an arbitrary number of Smart Waste Bin devices in a simulated environment with adjustable parameters. At the program start-up, a user-specified number of Smart Waste Bins devices are simulated, placed in an area of interest either randomly or in specific positions. Then, the simulation system runs the engine for the process of waste generation. We leveraged Python capabilities to generate a discrete-time simulation scenario that can either run in real-time or in a speed-up fashion. Each bin’s waste level at a certain time is modelled as a normal distribution, according to [45]. Indeed, the amount of waste deposited by each person in a bin can be represented as a stochastic variable. Therefore, according to the central limit theorem, the sum of many stochastic variables of arbitrary probability distributions approaches a normal distribution. The simulator allows to use five template distributions for each bin, according to different usage profiles from very low to very high usages, which in turn control the mean and variance of the associated normal distribution. Moreover, in order to adhere to real-world constraints, the simulator takes into consideration specific environment characteristics such as university closing time, holidays, and most expected waste type. Periodically, the data generated by each bin in the simulator is transmitted to a remote server via MQTT, using the same message format as the prototype. The smart waste bin simulation system is available at: https://tinyurl.com/SWB-sim (accessed on 22 May 2021).

### 5.2. Management Server

**Figure 13:**
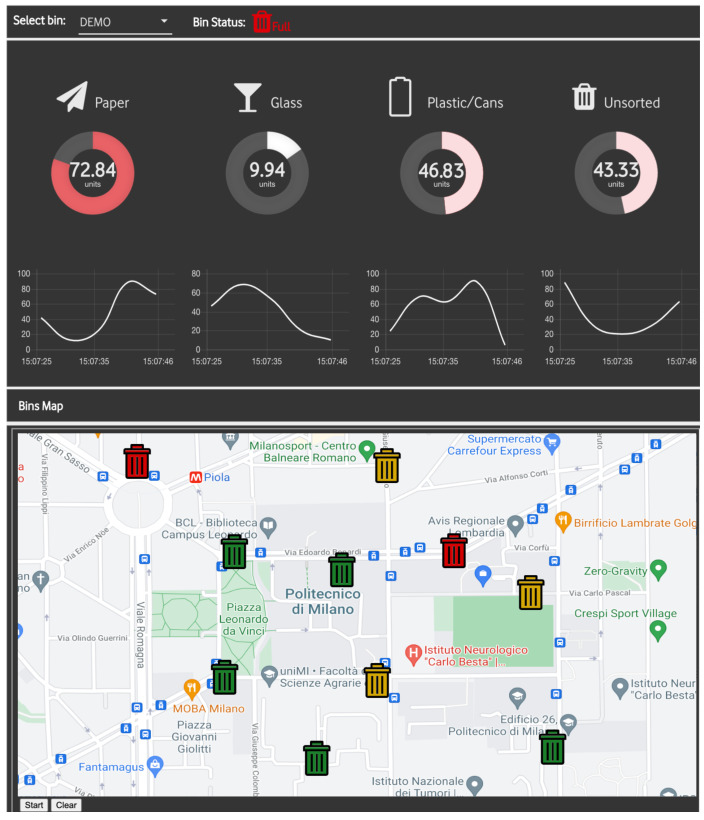
Smart waste bin backend dashboard implemented with Node-RED. The map shows the bins’ position as well as a graphical summary of the fill levels. Respectively, red when at least one class is greater than 70%; yellow when at least one class is greater than 40% and green when all the classes are below or equal 40%.

All data produced and transmitted by the SWB, real or simulated, are received by a server application running on a public server. The main tasks of the management server are the following:

1. *Data storage*: the server runs an MQTT broker that accepts messages from the smart waste bins. Each module publishes messages on specific MQTT topics: for example, in a scenario with two SWBs named swb1 and swb2, the topic smartbin/swb1/fle/glass is used for publishing messages of the glass bag’s filling levels; while the topic smartbin/swb2/daily is used for communicating the daily usage summary of the recycling bin as a Json file. Upon reception of a message, the server reads its content and saves the received information in a local SQL database.
2. *Data visualization*: the server also provides a web-based dashboard for data visualization and monitoring purposes. The dashboard is implemented with Node-RED, a framework built on top of Node.js that has recently become very popular in IoT application development. As shown in Figure 13, the dashboard shows aggregated information for each smart waste bin connected to the system: (i) on the top part, the fill levels for the four materials with their daily correspondent trend represented in a chart; and (ii) in the bottom part, a map summarizing the status of all the SWBs present in an area, with different colours according to the overall fill level of each bin, allowing to easily keep track of the status of the bin from the landfill operators.
3. *TSP for waste collection*: The management server also allows to calculate an optimized route for the operator in charge of the waste collection. The task is faced as a *Travelling Salesman Problem (TSP)*. In particular, the goal is to minimise the travelling time starting and finishing at a specific node (e.g., the landfill site) after visiting each other node exactly once. In particular, the nodes are represented by the bins and the weight on the links is the travel time of a specific road. Moreover, to avoid useless stops at an empty recycling bin, the SWB with a filling level lower than 75% of the total are automatically excluded by the TSP problem.

## 6. Conclusions

We have presented the design and implementation of a waste management system for Smart Campuses. The system is based on a smart waste bin prototype, an innovative device that can be used for automatically recycling objects using a hybrid sensor/image-based classifier. Results showed that the proposed approach reached an accuracy of over 97% for waste classification. In addition, we evaluated the device in different network scenarios, including moving the artificial intelligence on the MEC of the 5G network, reducing the recognition latency and the energy consumption. Moreover, we presented an application server which is able to easily monitor the status of waste bins present in the campus, as well as optimizing the management procedures (e.g., waste collection). We believe such a system will be extremely useful in the near future, considering the increased environmental impact of waste generated by people, which requires correct recycling. For this reason, we plan to create many other smart waste bin devices and deploy them on the university premises. This will also enable the possibility to study the interaction between students and smart waste bins, paving the way for possible future system optimizations.

## Figures and Tables

**Table 1:** List of objects contained in each waste class of the dataset.

**Glass**	Coke bottle, Beck’s Beer, Aperol Bottle Heineken Beer, Jar, Red Beer, Water Bottle
**Paper**	Business Card, Candy Box, Cup, Flyers Paper Bag, Juice Box, Magazine, Paper Napkins, Newspaper
**Plastic**	Blue Bottle, White Bottle, Green Bottle Coffee Capsule Packet, Transparent Glass White Dish, Green Dish, Red Dish Cutlery, Tea bottle, Fiesta Snack, Yogurt Cup, Plastic Bag
**Metal**	Aluminium can, Metal Box, Aluminium Foil, Jar Lid
**Unsorted**	Backing Paper, Bic Pen, CD, Cigarettes, Lighter, Marker, Receipt

**Table 2:** Summary table reporting the per class average and standard deviation obtained by the Inductive Sensor (IS), Capacitive Sensor (CS) and Photoelectric sensors (PS).

	IS	CS	PS
**Glass**	0 ± 0.0	0.96 ± 0.23	14.28 ± 8.9
**Paper**	0 ± 0.0	0.12 ± 0.11	0.68 ± 0.6
**Plastic**	0 ± 0.0	0.12 ± 0.12	17.00 ± 8.7
**Metal**	0.93 ± 0.25	0.98 ± 0.12	4.35 ± 2.3
**Unsorted**	0 ± 0.0	0.18 ± 0.13	7.12 ± 3.2

**Table 3:** Test confusion matrix obtained for sensor-based classification.

		Predicted Class				
**True** **Class**		**Glass**	**Paper**	**Plastic**	**Metal**	**Unsorted**
	**Glass**	530 (97%)	6 (1%)	2 (<1%)		8 (1%)
	**Paper**		697 (99%)			5 (1%)
	**Plastic**	1 (1%)	44 (4%)	846 (83%)		123 (12%)
	**Metal**	2 (1%)			310 (99%)	
	**Unsorted**	1 (<1%)	35 (6%)	96 (18%)		414 (76%)

**Table 4:** Test confusion matrix obtained for image-based classification.

		Predicted Class				
**True** **Class**		**Glass**	**Paper**	**Plastic**	**Metal**	**Unsorted**
	**Glass**	486 (89%)		39 (7%)		21 (4%)
	**Paper**	21 (3%)	653 (93%)	22 (3%)		6 (1%)
	**Plastic**	40 (4%)		963 (95%)		11 (1%)
	**Metal**	3 (1%)	4 (1%)		283 (91%)	22 (7%)
	**Unsorted**	11 (2%)	4 (1%)	6 (1%)	7 (1%)	518 (95%)

**Table 5:** Test confusion matrix obtained for hybrid classification with integration at prediction.

		Predicted Class				
**True** **Class**		**Glass**	**Paper**	**Plastic**	**Metal**	**Unsorted**
	**Glass**	535 (98%)	3 (<1%)	2 (<1%)	0	6 (<1%)
	**Paper**	2 (<1%)	700 (99%)			
	**Plastic**	7 (1%)	20 (2%)	951 (94%)		36 (3%)
	**Metal**	2 (<1%)			310 (99%)	
	**Unsorted**	11 (2%)	9 (2%)	11 (2%)	12 (2%)	503 (92%)

**Table 6:** Test confusion matrix obtained for hybrid classification with integration at learning.

		Predicted Class				
**True** **Class**		**Glass**	**Paper**	**Plastic**	**Metal**	**Unsorted**
	**Glass**	542 (99%)		2 (<1%)		2 (<1%)
	**Paper**		701 (99%)			1 (<1%)
	**Plastic**	1 (<1%)	14 (1%)	963 (95%)		36 (3%)
	**Metal**				312 (100%)	
	**Unsorted**	6 (1%)	7 (1%)	13 (3%)		520 (95%)

**Table 7:** Total waste recognition time.

	Local	Cloud Server	MEC Server
**Avg. Data Transfer Time (ms)**	-	343.3	191.3
**Avg. Classification Time (ms)**	3159.2	123.9	123.9
**Avg. Total Recognition Time (ms)**	3159.2	467.1	315.2

**Table 8:** Energy consumption of the Raspberry Pi when the waste classifier is run locally, on the MEC or on the cloud server.

	Local	Cloud Server	MEC Server
**Bin Energy Consumption (J/object)**	11.69	1.28	0.93

## Data Availability

The waste dataset and the Smart Waste Bin simulator used in this work are available at https://tinyurl.com/SWB-dataset and https://tinyurl.com/SWB-sim, (accessed on 22 May 2021).
